# Supplementary material for: Risk Prediction Models for Oral Cancer: A Systematic Review
Source: Cancers (Basel). 2024 Jan 31;16(3):617. doi: 10.3390/cancers16030617 (PMC10854942; doi:10.3390/cancers16030617)
Supplement: Supplementary file 1 [file cancers-16-00617-s001.zip › Supplementary File Table S1. Search Strategy.pdf]

Table S1.1. Search strategy for Medline.

	**Search line**
1	risk*.mp. or exp Risk/ or exp Risk Assessment/
2	chance*.mp.
3	exp Probability/ or likelihood*.mp. or exp Likelihood Functions/
4	1 or 2 or 3
5	predict*.mp.
6	exp Models, Theoretical/ or model*.mp. or exp Models, Statistical/
7	score*.mp.
8	5 or 6 or 7
9	exp Mouth Neoplasms/ or (exp Squamous Cell Carcinoma of Head/ and Neck/)
10	(((oral or mouth or oral cavit* or tongue* or lip or lips or gingiva* or palatal or salivary gland* or oropharyn*) adj3 (cancer* or neoplas* or tumo?r* or carcinom*)) or oral squamous cell carcinoma).ti,ab.
11	9 or 10
12	4 and 8 and 12
13	Review/
14	Letter/
15	Editorial/
16	Comment/
17	13 or 14 or 15 or 16
18	12 not 17
19	limit 18 to human
20	limit 19 to dt=19460101-20221130

Table S1.2. Search strategy for Embase.

	**Search line**
1	exp cancer risk/ or risk*.mp. or exp risk/ or exp risk factor/ or exp risk assessment/
2	chance*.mp.
3	exp probability/ or likelihood*.mp.
4	1 or 2 or 3
5	exp prediction/ or predict*.mp.
6	exp mathematical model/ or model*.mp. or exp model/ or exp statistical model/
7	score.mp.
8	5 or 6 or 7
9	exp mouth cancer/ or exp mouth squamous cell carcinoma/
10	(((oral or mouth or oral cavity or tongue* or lip or lips or gingiva* or palatal or salivary gland* or oropharyn*) adj3 (cancer* or neoplas* or tumo?r* or carcinom*)) or mouth squamous cell carcinoma).ti,ab.
11	9 or 10
12	4 and 8 and 11
13	review.pt.
14	letter.pt.
15	editorial.pt.
16	13 or 14 or 15
17	12 not 16
18	limit 17 to human
19	limit 18 to dc=19740101-20221130

Table S1.3. Search strategy for Cochrane Library.

	Search line
#1	(risk*):ti,ab,kw
#2	MeSH descriptor: [Risk] explode all trees
#3	MeSH descriptor: [Risk Assessment] explode all trees
#4	MeSH descriptor: [Likelihood Functions] explode all trees
#5	(chance):ti,ab,kw
#6	MeSH descriptor: [Probability] explode all trees
#7	(likelihood):ti,ab,kw
#8	#1 or #2 or #3 or #4 or #5 or #6 or #7
#9	(predict*):ti,ab,kw
#10	MeSH descriptor: [Models, Statistical] explode all trees
#11	MeSH descriptor: [Models, Theoretical] explode all trees
#12	(model*):ti,ab,kw
#13	(score*):ti,ab,kw
#14	#9 or #10 or #11 or #12 or #13
#15	MeSH descriptor: [Mouth Neoplasms] explode all trees
#16	MeSH descriptor: [Squamous Cell Carcinoma of Head and Neck] explode all trees
#17	("oral squamous-cell carcinoma*" or "oral squamous cell carcinoma"):ti,ab,kw
#18	((oral or mouth or oral cavit* or tongue* or lip or lips or gingiva* or palatal or salivary gland* or oropharyn*) and (cancer* or neoplas* or tumo?r* or carcinom*)):ti,ab,kw
#19	#15 or #16 or #17 or #18
#20	#8 and #14 and #19
#21	#20 with Cochrane Library publication date to Nov 2022, in Trials
