# Supplementary material for: Risk Prediction Models for Oral Cancer: A Systematic Review
Source: Cancers (Basel). 2024 Jan 31;16(3):617. doi: 10.3390/cancers16030617 (PMC10854942; doi:10.3390/cancers16030617)
Supplement: Supplementary file 1 [file cancers-16-00617-s001.zip › Supplementary File Table S2. Extraction Form.pdf]

Table S2. Extracted items from individual models.

Domain	Key items	Details
Study Description & Development	First Author	
	Year	
	Journal	
	Title	
	Study Dates	
	Study Settings	
	Country, Region	
	Type of Population	
	Type of Model	
	Source of Data	
	Data Collection Method	
	Model Classification (TRIPOD guidelines)	
	Model Performance: Reference for validation studies - First author (year)	
	Study Design	
	Outcome	Lip
		Oral cavity (tongue, buccal, palate, gum)
		Oropharynx
	Diagnostic/Prognostic	Diagnostic
		Prognostic
Participants: Case-control (Model Development Study)	Methods of selection of population	
	Selection of cases	
	Selection of controls	
	Exclusion criteria	
	Participant description	
Participants: Cohort (Model Development Study)	Methods of selection of population	
	Cohort description	
	Follow-up duration	
	Exclusion criteria	
	Participant description	
Participants: Cross-sectional (Model Development Study)	Methods of selection of population	
	Description of study participants	
	Exclusion criteria	
	Participant description	
Participants: Other study types (Model Development Study)	Methods of selection of population	
	Sample size	
	Notes on study participants	
Participant Size & Age (Model Development Study)	Total Population	No. Eligible (male sex, %)
		No. Included (male sex, %)
		Age (mean ± SD)
		Age (median ± CI)
	Cases	No. Eligible (male sex, %)
		No. Included (male sex, %)
		Age (mean ± SD)
		Age (median ± CI)
		Age (intervals)
	Controls	No. Eligible (male sex, %)
		No. Included (male sex, %)
		Age (mean ± SD)
		Age (median ± CI)
		Age (intervals)
Missing Data	Number of participants with any missing value (include predictors and outcomes)	
	Number of participants with missing data for each predictor	
	Handling of missing data (e.g., complete-case analysis, imputation)	
List of Predictors	Age	Considered
		Included
		Details
	Gender	Considered
		Included

Domain	Key items	Details
		Details
	Ethnicity	Considered
		Included
		Details
	Family History	Considered
		Number of conditions considered as separate predictors
		Included
		Number of conditions included as separate predictors
		Details
	Weight/BMI	Considered
		Included
		Details
	Betel Quid Chewing	Considered
		Included
		Details
	Smoking (Status)	Considered
		Included
		Details
	Alcohol Consumption	Considered
		Included
		Details
	Diet (meat/fish etc.)	Considered
		Number considered
		Included
		Number Included
		Details
	Physical Activity	Considered
		Included
		Details
	Biomarkers	Considered
		Number considered
		Included
		Number Included
		List biomarkers used
		Details
	Genetic Factors	Considered
		Number Considered
		Included
		Number Included
		List genetic factors used
		Details
	Oral Health	Considered
		Included
		Details
	UV Light Exposure	Considered
		Included
		Details
	HPV	Considered
		Included
		Details
	Mouthwash Use	Considered
		Included
		Details
	Denture Irritation	Considered
		Included
		Details
	Previous Cancer Diagnosis	Considered
		Number of cancers considered as separate predictors
		Included

Domain	Key items	Details
		Number of cancers included as separate predictors
		Details
	Other	Considered
		Number Considered
		Included
		Number Included
		Details
Outcome(s)	Outcome measure: Definition and measurement method	
	Was the same outcome definition (and method for measurement) used in all patients?	
	Was the outcome assessed without knowledge of the candidate predictors (i.e., blinded)?	
	Were candidate predictors part of the outcome (e.g., in panel or consensus diagnosis)?	
	Time of outcome occurrence or summary of duration of follow-up	
Predictors	Predictors (included)	
	Definition and method for measurement of candidate predictors	
	Timing of predictor measurement (e.g., at screening)	
	Were predictors assessed blinded for outcome, and for each other (if relevant)?	
	Handling of predictors in the modelling (e.g., continuous, linear, non-linear transformations or categorised)	
	Predictors (considered but not included)	
Model Development	Models of interest	
	Selection of predictors for consideration	
	Selection of predictors for the modelling	
	Modelling method (e.g., logistic, survival, neural network)	
	Methods of administration	
Model Performance	Performance measures summary	
	Discrimination	AUROC
		C-statistic
		Other
	Calibration	χ^2 test
		Goodness-of-fit
		Likelihood ratio test
		Other
	Accuracy	Sensitivity
		Specificity
		PPV
		NPV
	Other	
Model Evaluation	Was the model adjusted or updated in case of poor validation?	
Internal Validation	Does the study include internal validation?	
	Type of internal validation	
	Internal Validation Population	
	Methods of selection of population	
	Total Population	No. Eligible (male sex, %)
		No. Included (male sex, %)
		Age (mean ± SD)
		Age (median ± CI)
	Cases	No. Eligible (male sex, %)
		No. Included (male sex, %)
		Age (mean ± SD)
		Age (median ± CI)
		Age (intervals)
	Controls	No. Eligible (male sex, %)
		No. Included (male sex, %)
		Age (mean ± SD)
		Age (median ± CI)
		Age (intervals)

Domain	Key items	Details
	Performance measures summary	
	Discrimination	AUROC
		C-statistic
		Other
	Calibration	χ^2 test
		Goodness-of-fit
		Likelihood ratio test
		Other
	Accuracy	Sensitivity
		Specificity
		PPV
		NPV
	Other	
	Missing data	Number of participants with any missing value (include predictors and outcomes)
		Number of participants with missing data for each predictor
		Handling of missing data (e.g., complete-case analysis, imputation)
	Was the model adjusted or updated in case of poor validation?	
External Validation	Does the study include external validation?	
	Type of external validation	
	Methods of selection of population	
	External Validation Study	Study Dates
		Study Settings
		Country, Region
		Type of Population
		Source of Data
		Data Collection Method
		Study Design
	External Validation Population	Selection of cases
		Selection of controls
		Exclusion criteria
		Participant description
	Total Population	No. Eligible (male sex, %)
		No. Included (male sex, %)
		Age (mean ± SD)
		Age (median ± CI)
	Cases	No. Eligible (male sex, %)
		No. Included (male sex, %)
		Age (mean ± SD)
		Age (median ± CI)
		Age (intervals)
External Validation	Controls	No. Eligible (male sex, %)
		No. Included (male sex, %)
		Age (mean ± SD)
		Age (median ± CI)
		Age (intervals)
	Performance measures summary	
	Discrimination	AUROC
		C-statistic
		Other
	Calibration	χ^2 test
		Goodness-of-fit
		Likelihood ratio test
		Other
	Accuracy	Sensitivity
		Specificity
		PPV
		NPV
	Other	

Domain	Key items	Details
	Missing Data	Number of participants with any missing value (include predictors and outcomes)
		Number of participants with missing data for each predictor
		Handling of missing data (e.g., complete-case analysis, imputation)
	Was the model adjusted or updated in case of poor validation?	
Results, Interpretation, and Discussion	Final and other multivariable models presented, including predictor weights or regression coefficients, intercept, baseline survival, model performance measures	
	Alternative presentation of the final prediction models	
	Comparison of the distribution of predictors (including missing data) for development and validation datasets	
	Interpretation of presented models	
	Comparison with other studies (generalisability, strengths, and limitations)	
