# Supplementary material for: Risk Prediction Models for Oral Cancer: A Systematic Review
Source: Cancers (Basel). 2024 Jan 31;16(3):617. doi: 10.3390/cancers16030617 (PMC10854942; doi:10.3390/cancers16030617)
Supplement: Supplementary file 1 [file cancers-16-00617-s001.zip › Supplementary File Table S3. List of Excluded Articles.pdf]

Table S3.1. Articles not in English Language (n = 10).

First author	Title
Andreotti	Occupational status and cancer of the oral cavity and oropharynx.
Bao	Association of single nucleotide polymorphisms of TBX5 gene and environmental exposure index with susceptibility to oral cancer.
Li	Association between dietary fatty acid patterns and risk of oral cancer by principal component analysis.
Su	Relationship between medium-chain saturated fatty acids and risk of oral cancer.
Toporcov	Consumption of animal-derived foods and mouth and oropharyngeal cancer.
Wen	Spicy food consumption and risk of lip, oral cavity and pharynx cancers: A prospective cohort study of Chinese adults.
Wu	A case-control study: association between oral hygiene and oral cancer in non-smoking and non-drinking women.
Yan	Tea, coffee intakes and risk of oral squamous cell carcinoma: a case-control study.
Yuan	Association between genetic variants in microRNA biosynthesis genes and the risk of head and neck squamous cell carcinoma.
Zhang	Correlation of the polymorphism of EC-SOD and GSTM1 and smoking with oral cancer risk.

Table S3.2. Articles not predicting the risk of developing oral cancer (n = 73).

First author	Title
Adeeba	A comparative profiling of oral cancer patients and high risk niswar users using FT-IR and chemometric analysis
Adeoye	Performance of a simplified scoring system for risk stratification in oral cancer and oral potentially malignant disorders screening
Amarasinghe	Derivation and validation of a risk-factor model for detection of oral potentially malignant disorders in populations with high prevalence
Anderson	Biologic predictors of serologic responses to HPV in oropharyngeal cancer: The HOTSPOT study
Antonsson	Variants of EVER1 and EVER2 (TMC6 and TMC8) and human papillomavirus status in patients with mucosal squamous cell carcinoma of the head and neck
Banavar	The salivary metatranscriptome as an accurate diagnostic indicator of oral cancer
Boeing	Intake of fruits and vegetables and risk of cancer of the upper aero-digestive tract: the prospective EPIC-study
Bradshaw	Associations Between Dietary Patterns and Head and Neck Cancer The Carolina Head and Neck Cancer Epidemiology Study
Chen	CYP26A1 Is a Novel Biomarker for Betel Quid-Related Oral and Pharyngeal Cancers
Chen	Genetic and Proteinic Linkage of MAO and COMT with Oral Potentially Malignant Disorders and Cancers of the Oral Cavity and Pharynx
Chen	Gene expression profiling identifies genes predictive of oral squamous cell carcinoma
Chien	Polygenic Panels Predicting the Susceptibility of Multiple Upper Aerodigestive Tract Cancer in Oral Cancer Patients
Dahlstrom	Diagnostic accuracy of serum antibodies to human papillomavirus type 16 early antigens in the detection of human papillomavirus-related oropharyngeal cancer
Dalmartello	Dietary patterns and oral and pharyngeal cancer using latent class analysis
Datzmann	Outdoor air pollution, green space, and cancer incidence in Saxony: a semi-individual cohort study
Deschasaux	Prospective associations between Vitamin D status, Vitamin D-related gene polymorphisms, and risk of tobacco-related cancers
Downer	Evaluation of screening for oral cancer and precancer in a company headquarters
Edefonti	Natural vitamin C intake and the risk of head and neck cancer: A pooled analysis in the International Head and Neck Cancer Epidemiology Consortium
Ghosh	Deep reinforced neural network model for cyto-spectroscopic analysis of epigenetic markers for automated oral cancer risk prediction
Gleber-Netto	Salivary Biomarkers for Detection of Oral Squamous Cell Carcinoma in a Taiwanese Population
Graff	Cross-cancer evaluation of polygenic risk scores for 16 cancer types in two large cohorts
Habbous	The changing incidence of human papillomavirus-associated oropharyngeal cancer using multiple imputation from 2000 to 2010 at a Comprehensive Cancer Centre
Han	Reduced mRNA expression of nucleotide excision repair genes in lymphocytes and risk of squamous cell carcinoma of the head and neck

First author	Title
Hang	KIT polymorphisms were associated with the risk for head and neck squamous carcinoma in Chinese population
Hardcastle	Empirical Bayesian analysis of paired high-throughput sequencing data with a beta-binomial distribution
Hippisley-Cox	Development and validation of risk prediction algorithms to estimate future risk of common cancers in men and women: prospective cohort study
Jain	Identification of potential salivary biomarker panels for oral squamous cell carcinoma
Jayasekara	Lifetime alcohol consumption and upper aero-digestive tract cancer risk in the Melbourne Collaborative Cohort Study
Jin	Genetic variation in MDM2 and p14ARF and susceptibility to salivary gland carcinoma
Jourenkova-Mironova	High-activity microsomal epoxide hydrolase genotypes and the risk of oral, pharynx, and larynx cancers
Kachuri	Pan-cancer analysis demonstrates that integrating polygenic risk scores with modifiable risk factors improves risk prediction
Langevin	CpG island methylation profile in noninvasive oral rinse samples is predictive of oral and pharyngeal carcinoma
Lee	Genetically lowered microsomal epoxide hydrolase activity and tobacco-related cancer in 47,000 individuals
Li	Serum circulating human mRNA profiling and its utility for oral cancer detection
Lim	The Performance of an Oral Microbiome Biomarker Panel in Predicting Oral Cavity and Oropharyngeal Cancers
Liu	Metabolic dysfunction-associated fatty liver disease and the risk of 24 specific cancers
Liu	Apoptotic capacity and risk of squamous cell carcinoma of the head and neck
Liyanage	Promoter hypermethylation of tumor-suppressor genes p16^{ink4a}, rassf1a, timp3, and pcqap/med15 in salivary dna as a quadruple biomarker panel for early detection of oral and oropharyngeal cancers
Lotfi	Serum Level of Interleukin-6 in Patients with Oral Tongue Squamous cell Carcinoma
Mainous	A Practical Risk Measure for Identification of Adults With Oncogenic Oral Human Papillomavirus: Potential Use in Primary Care
Matsuo	Folate, alcohol, and aldehyde dehydrogenase 2 polymorphism and the risk of oral and pharyngeal cancer in Japanese
Matullo	DNA repair polymorphisms and cancer risk in non-smokers in a cohort study
Mayne	Cross validated serum small extracellular vesicle microRNAs for the detection of oropharyngeal squamous cell carcinoma
Ni	Associations of hypoxia inducible factor-1alpha gene polymorphisms with susceptibility to digestive tract cancers: a case-control study and meta-analysis
Nishimoto	Cyclin D1 gene polymorphism as a risk factor for squamous cell carcinoma of the upper aerodigestive system in non-alcoholics
Olshan	GSTM1, GSTT1, GSTP1, CYP1A1, and NAT1 polymorphisms, tobacco use, and the risk of head and neck cancer
Oze	Comparison between self-reported facial flushing after alcohol consumption and ALDH2 Glu504Lys polymorphism for risk of upper aerodigestive tract cancer in a Japanese population
Paiva	Cytological Screening Model of Normal Oral Mucosa Exposed to Carcinogens: A Pilot Study
Parfenova	An improved algorithm using a Health Canada-approved DNA-image cytometry system for non-invasive screening of high-grade oral lesions
Park	Epoxide hydrolase genotype and orolaryngeal cancer risk: interaction with GSTM1 genotype
Pattani	Endothelin Receptor Type B Gene Promoter Hypermethylation in Salivary Rinses Is Independently Associated with Risk of Oral Cavity Cancer and Premalignancy
Pereira	Salivary markers and risk factor data: A multivariate modeling approach for head and neck squamous cell carcinoma detection
Perez-Sayans	Measurement of ATP6V1C1 expression in brush cytology samples as a diagnostic and prognostic marker in oral squamous cell carcinoma
Poell	Oral cancer prediction by noninvasive genetic screening
Radunovic	The MMP-2 and MMP-9 promoter polymorphisms and susceptibility to salivary gland cancer
Rahman	Histopathologic Oral Cancer Prediction Using Oral Squamous Cell Carcinoma Biopsy Empowered with Transfer Learning
Randhawa	Integrated network analysis and logistic regression modeling identify stage-specific genes in Oral Squamous Cell Carcinoma
Rêgo	Development and professional validation of an App to support Oral Cancer Screening
Ren	Circulating high mobility group AT-hook 2 and pleomorphic adenoma gene 1 in blood of patients with oral squamous cell carcinoma

First author	Title
Sahu	Oral cancer screening: serum Raman spectroscopic approach
Schussel	EDNRB and DCC Salivary Rinse Hypermethylation Has a Similar Performance as Expert Clinical Examination in Discrimination of Oral Cancer/Dysplasia versus Benign Lesions
Shah	Uncovering the potential of CD44v/SYNE1/miR34a axis in salivary fluids of oral cancer patients
Sharma	Usage of Probabilistic and General Regression Neural Network for Early Detection and Prevention of Oral Cancer
Sohn	Machine Learning Based Radiomic HPV Phenotyping of Oropharyngeal SCC: A Feasibility Study Using MRI
Sturgis	Polymorphisms of DNA repair gene XRCC1 in squamous cell carcinoma of the head and neck
Tseng	Development and validation of machine learning-based risk prediction models of oral squamous cell carcinoma using salivary autoantibody biomarkers
Wu	Epistasis of oxidative stress-related enzyme genes on modulating the risks in oral cavity cancer
Wyss	Single-nucleotide polymorphisms in nucleotide excision repair genes, cigarette smoking, and the risk of head and neck cancer
Yap	Non-invasive screening of a microRNA-based dysregulation signature in oral cancer and oral potentially malignant disorders
Yin	Effects of in IL-1B/IL-1RN variants on the susceptibility to head and neck cancer in a chinese Han population
Yu	Association between Single Nucleotide Polymorphisms in ERCC4 and Risk of Squamous Cell Carcinoma of the Head and Neck
Yu	Saliva protein biomarkers to detect oral squamous cell carcinoma in a high-risk population in Taiwan
Zheng	Tobacco carcinogen-detoxifying enzyme UGT1A7 and its association with orolaryngeal cancer risk

Table S3.3. Articles publishing models without a combination of two or more risk factors (n = 8).

First author	Title
Chang	Investigating the association between oral hygiene and head and neck cancer
Mascharak	Detecting oropharyngeal carcinoma using multispectral, narrow-band imaging and machine learning
Narayanan	The Utility of Beta 2 Microglobulin (B2M) as an Initial Diagnostic Tool for Oral Squamous Cell Carcinoma (OSCC): Evidence from a Malaysian Scenario
Shieh	Lack of Salivary Long Non-Coding RNA XIST Expression Is Associated with Increased Risk of Oral Squamous Cell Carcinoma: A Cross-Sectional Study
Su	Oral microbial dysbiosis and its performance in predicting oral cancer
Tsai	Contribution of MMP2 Promoter Genotypes to Oral Cancer Susceptibility, Recurrence and Metastasis in Taiwan
Wang	Human leucocyte antigen-G 14-bp InDel polymorphism and oral squamous cell carcinoma risk in Chinese Han population: A case-control study
Yokoyama	Multiple cancers associated with esophageal and oropharyngolaryngeal squamous cell carcinoma and the aldehyde dehydrogenase-2 genotype in male Japanese drinkers

Table S3.4. Articles publishing models not applicable in the general population (n = 13).

First author	Title
Alhazmi	Application of artificial intelligence and machine learning for prediction of oral cancer risk
Bharti	Functional genetic variants of CTLA-4 and risk of tobacco-related oral carcinoma in high-risk North Indian population
Chen	Application of simulation-based CYP26 SNP-environment barcodes for evaluating the occurrence of oral malignant disorders by odds ratio-based binary particle swarm optimization: A case-control study in the Taiwanese population
Drake	Timing, number, and type of sexual partners associated with risk of oropharyngeal cancer
Hung	Assessment of the Risk of Oral Cancer Incidence in A High-Risk Population and Establishment of A Predictive Model for Oral Cancer Incidence Using A Population-Based Cohort in Taiwan
Mitra	Risk assessment of p53 genotypes and haplotypes in tobacco-associated leukoplakia and oral cancer patients from eastern India
Notani	Role of Alcohol in Cancers of the Upper Alimentary-Tract - Use of Models in Risk Assessment
Pereira	Risk Stratification System for Oral Cancer Screening

First author	Title
Solomon	Polymorphism in ADH and MTHFR genes in oral squamous cell carcinoma of Indians
Sunny	A smart tele-cytology point-of-care platform for oral cancer screening
Tewari	Estimating the conditional probability of developing human papilloma virus related oropharyngeal cancer by combining machine learning and inverse Bayesian modelling
Wang	Machine Learning Based Risk Prediction Models for Oral Squamous Cell Carcinoma Using Salivary Biomarkers
Yokoyama	Alcohol-related cancers and aldehyde dehydrogenase-2 in Japanese alcoholics
