# Supplementary material for: Risk Prediction Models for Oral Cancer: A Systematic Review
Source: Cancers (Basel). 2024 Jan 31;16(3):617. doi: 10.3390/cancers16030617 (PMC10854942; doi:10.3390/cancers16030617)
Supplement: Supplementary file 1 [file cancers-16-00617-s001.zip › Supplementary File Table S4. Summary of development and validation studies.pdf]

Summary of development and validation studies

Table S4.1. Details of development studies.

First author, year	Study setting[a], type[b]	Country	Selection of risk factors	Study dates	Follow-up duration or time of outcome occurrence	Data collection methods	Selection of cases	Selection of control	Selection of cohort	Exclusion criteria	Cases and controls (% male)	Age, mean ± SD or median (IQR)
**Development studies**												
Antunes, 2013	H, CC	Brazil	Literature	1998–2008	No information	Interview, medical record	Histologically confirmed oral or oropharyngeal cancer (C01-C06, C09, C10)	Individuals in outpatient units with the same referral routes as case patients	-	Cases: Stage 0 cancers, C00, C11, C13, C14 Controls: Individuals with drinking and smoking-related diseases and aerodigestive tract diseases	1,144 (80.7%) and 1,661 (73.2%)	No information
Bao, 2020a	H, CC	China	Literature	2011–2018	No information	Interview-based questionnaire, blood sample, biopsy	Primary oral cancer based on WHO classification (C01-C06)	Recruited patients with acute non-neoplastic conditions without history of malignant diseases	-	All participants: no whole blood sample and no qualified DNA quality Cases: History of chemotherapy and raditotherapy Controls: History of malignant disease	235 (60.4%) and 406 (40.9%)	Cases: 58.40 ± 12.99 Controls: 58.81 ± 7.13
Chen, 2017	HM, CC	China	No information	2010–2016	No information	Interview (using structured questionnaire)	Newly diagnosed oral cancer patients at the hospital	Individuals without history of cancer or cancer-related diseases from the same hospital and community population	-	Cases: Second primary oral cancer, recurrent or metastasised oral cancer; previous radio- or chemotherapy Controls: Individuals with diseases that require changes in diet	930 (63.23%) and 2,667 (63.3%)	No information
Chen, 2018 (a, b)	H, CC	China	Literature	2010–2017	No information	Interview (using structured questionnaire)	Newly diagnosed oral cancer patients at the hospital	Age frequency-matched individuals without history of cancer or cancer-related diseases from the same hospital	-	Recurrent or metastatic oral cancer	2018a: 616 (100%) and 1,308 (100%) 2018b: 362 (0%) and 1,338 (0%)	No information
Chen, 2022	H, CC	China	Literature	2010–2018	No information	Interview, questionnaires, blood sample	Histologically confirmed primary oral	Age and gender frequency-matched to	-	Recurrent or metastasized cancer; previous	325 (62.2%) and 648 (67.1%)	Cases: 61.5 ± 12.5 Controls: 62.4 ±

						collection	cancer; Chinese Han ethnicity and resides in Fujian province; aged 20–80 years	cases from the same hospital in the same period		chemotherapy or radiotherapy treatment		10.5
Cheung, 2021	M, Cohort	India	*A priori* selection	1996–1998, 1999–2001, 2002–2004	7 years	Household survey, oral visual inspections by trained health workers, clinical examination, biopsy, medical record, interview	-	-	Apparently healthy individuals aged 35 or over in the general population in a cluster RCT with oral screening as an intervention	Individuals with a history of oral cancer	Control arm: 95,354 (44%) Screening arm: 96,516 (43%)	Control arm: 45 (38–57) Screening arm: 45 (37–57)
He, 2021 (a, b)	H, CC	China	Literature	2011-2018	No information	Interview-based questionnaire, blood samples	Primary oral cancer	Healthy individuals without history of cancer from the hospital	-	Cases: recurrent or metastasised oral cancer; radio- or chemotherapy, history of severe systemic diseases, long-term use of dietary supplements Controls: different period of admission with case group, not from single households, not residing in Fujian or aged below 20 or over 80, long-term use of dietary supplements	2021a and 2021b: 324 (62.3%) and 650 (67.1%)	No information
Rao, 2016 (a, b)	H, CC	India	Literature	2011–2012	No information	Interview, medical record, oral examination	Histopatholo-gically confirmed oral cancer	Unmatched individuals without history of cancer from the same hospital	-	Individuals with cancer, or relatives/carers/ visiitors of cancer patients	180 (79.4%) and 272 (54.8%)	No information
Lee, 2020 (a-d)	HM, CC	United States	Literature	1981-2010	20 years	Interview, questionnaires	Individuals with invasive cancer of oral cavity, oropharynx, hypopharynx or larynx.	Age and sex frequency-matched	-	Individuals with cancers of the major salivary glands or of the nasal cavity/ ear/para-nasal sinuses	2020a: 1,554 (100%) and 6,864 (100%) 2020b: 830 (0%) and 3,437 (0%) 2020c: 2,167 (100%) and 6864 (100%) 2020d: 653 (0%) and 3,437 (0%)	No information
Tota, 2019	HG, CC	United	*A priori* selection	2009–2014	1 year	Interview (using	Newly diagnosed	Individuals aged	-	No information	2019a and 2019b:	Unweighted:

(a, b)		States		(control) and 2011–2015 (case)		computer-assisted self-interview), clinical data	OPC (the base of the tongue, soft palate, palatine tonsils and posterior pharyngeal wall) during 2011-2015	30-69 participating in the National health and Nutrition Examination Survey (NHANES) in 2009–2014			Unweighted: 241 (85.9%) and Weighted: 10,633 (82.5%) and	Cases: Unweighted: 57.3 (range: 31-78); Weighted: Cases: 57.1 (range: 31-69)
**Development studies, model incorporating genetic variants**												
Bao, 2020b	H, CC	China	Genetic variations in selenoprotein genes followed by genotype detection and Hardy-Weinberg Equilibrium	2011–2018	No information	Interview-based questionnaire, blood sample, biopsy	Primary oral cancer based on WHO classification (C01-C06)	Recruited patients with acute non-neoplastic conditions without history of malignant diseases	-	All participants: no whole blood sample and no qualified DNA quality<br>Cases: History of chemotherapy and raditotherapy<br>Controls: History of malignant disease	235 (60.4%) and 406 (40.9%)	Cases: 58.40 ± 12.99<br>Controls: 58.81 ± 7.13
Chung, 2017	H, CC	Taiwan	HapMap database followed by logistic regression	No information	No information	Interview, medical record, blood sample (DNA extraction)	Diagnosed OSCC	Gender and geographic area frequency-matched individuals with cataract and glaucoma, bone fractures and physical check-up	-	No information	Total population: 447 (96.2%) and 580 (97.4%)<br>Development population: 285 and 331	Total population:<br>Cases: 53.8 ± 13.7<br>Controls: 51.2 ± 10.4
Chung, 2019	H, CC	Taiwan	Somatic mutations reported in the Cancer Genome Atlas followed by logistic regression	No information	No information (Using age as follow-up years)	Interview	Newly diagnosed patients with OSCC	Patients with cataract and glaucoma, bone fractures and physical check-up from the same two hospitals	-	No information	360 (95.3%) and 486 (97.7%)	Cases: 54.2 ± 10.2<br>Controls: 51.7 ± 13.4
Fritsche, 2020 (a, b)	G, CC	United Kingdom	UK Biobank GWAS PheCode (2020a) and FINNGEN (2020b), GWAS summary statistics	No information	No information	GWAS summary statistics and genetic data from the UK Biobank	Diagnosis of cancer of the mouth (2020a) or tongue (2020b) in white European ancestry	Birth year matching with 9.9 control subjects per case	-	Non-European ancestry; entries with missing risk alleles, risk allele frequencies or SNP-disease OR	No information	No information
Miao, 2016	HG, CC	China	Previous literature on polymorphisms	2009–2013	No information	Interview-based questionnaire, blood sample	Newly diagnosed and histopatholo-gically confirmed OSCC	Age and sex-matched cancer-free individuals from the community	-	Individuals with secondary HNSCC or metastasis	462 and 1,552 (63.6%)	No information

Abbreviations: GWAS, genome-wide association studies; HNSCC, Head and neck squamous cell carcinoma; OCC, oral cavity cancer; OPC, oropharyngeal cancer; OR, odds ratio; OSCC, oral squamous cell carcinoma; SNP, single nucleotide polymorphism

[a]Study settings: G, general population; H, hospital-based; HG, a combination between hospital-based cases and population-based controls; HM, a combination between hospital-based cases and mixed (hospital- and population-

based) controls
[b]Study types: CC, case-control; Cohort

Table S4.2. Details of internal and external validation studies.

First author, year	Validation methods	Study setting[a], type[b]	Country	Selection of risk factors	Study dates	Follow-up duration or time of outcome occurrence*	Data collection methods	Selection of cases	Selection of control	Selection of cohort	Exclusion criteria	Differences with development population	Cases and controls (% male)	Age, mean ± SD or median (IQR)
**Internal validation studies**														
Chen, 2018 (a, b)	Bootstrapping with 1,000 resamples	HM, CC	China	Literature	2010–2017	No information	Interview (using structured questionnaire)	Newly diagnosed oral cancer patients at the hospital	Age frequency-matched individuals without history of cancer or cancer-related diseases from the same hospital	-	Recurrent or metastatic oral cancer	-	2018a: 616 (100%) and 1,308 (100%) 2018b: 362 (0%) and 1,338 (0%)	No information
Cheung, 2021	Five-fold cross-validation	G, Cohort	India	*A priori* selection	1996–1998, 1999–2001, 2002–2004	7 years	Household survey, oral visual inspections by trained health workers, clinical examination, biopsy, medical record, interview	-	-	Apparently healthy individuals aged 35 or over in the general population in a cluster RCT with oral screening as an intervention	Individuals with a history of oral cancer	-	Control arm: 95,354 (44%) Screening arm: 96,516 (43%)	Control arm: 45 (38–57) Screening arm: 45 (37–57)
Lee, 2020 (a-d)	Random split-sample	HM, CC	United States	Literature	1981–2010	20 years	Interview, questionnaires	Individuals with invasive cancer of oral cavity, oropharynx, hypopharynx or larynx.	Age and sex frequency-matched	-	Individuals with cancers of the major salivary glands or of the nasal cavity/ ear/para-nasal sinuses	The database was randomly split into 70% development set and 30% validation set	2020a: 1,554 (100%) and 6,864 (100%) 2020b: 830 (0%) and 3,437 (0%) 2020c: 2,167 (100%) and 6864 (100%) 2020d: 653 (0%) and 3,437 (0%)	No information
Rao, 2016 (a, b)	Bootstrapping with 200 resamples	H, CC	India	Literature	2011–2012	No information	Interview, medical record, oral examination	Histopatholo-gically confirmed oral cancer	Unmatched individuals without history of cancer from the same hospital	-	Individuals with cancer, or relatives/care rs/ visiitors of cancer patients	-	180 (79.4%) and 272 (54.8%)	No information
Tota, 2019 (a, b)	Non-random-split sample	HG, CC	United States	*A priori* selection	2009–2014 (control) and 2011–2015 (case)	1 year	Interview using computer-assisted self-interview, clinical data	Newly diagnosed OPC during 2011–2015	Individuals aged 30–69 participating in the NHANES survey in 2009–2014	-	No information	The last one-third recruited OPC cases	Total population: Unweighted: 241 (85.9%) and Weighted: 10,633 (82.5%) and	Unweighted: Cases: Unweighted: 57.3 (range: 31-78); Weighted: Cases: 57.1 (range: 31-

First author, year	Validation methods	Study setting[a], type[b]	Country	Selection of risk factors	Study dates	Follow-up duration or time of outcome occurrence*	Data collection methods	Selection of cases	Selection of control	Selection of cohort	Exclusion criteria	Differences with development population	Cases and controls (% male)	Age, mean ± SD or median (IQR)
													Validation population: 80 OPC cases	69)
**Internal validation studies, models incorporating genetic variants**														
Chung, 2017	Non-random-split sample	H, CC	Taiwan	HapMap database followed by logistic regression	No information	No information	Interview, medical record, blood sample (DNA extraction)	Diagnosed OSCC	Gender and geographic area frequency-matched individuals with cataract and glaucoma, bone fractures and physical check-up	-	No information	OSCC cases recruited in a different hospital	Total population 447 (96.2%) and 580 (97.4%) Validation population: 162 and 249	Total population: Cases: 53.8 ± 13.7 Controls: 51.2 ± 10.4
Fritsche, 2020 (a, b)	Random-split sample, cross-validation	G, CC	United Kingdom	Fixed p-value threshold UK Biobank GWAS PheCode (2020a) and FINNGEN (2020b)	No information	No information	GWAS summary statistics and genetic data from the UK Biobank	Diagnosis of cancer of the mouth (2020a) or tongue (2020b) in white European ancestry	Birth year matching with 9.9 control subjects per case	-	Non-European ancestry; entries with missing risk alleles, risk allele frequencies or SNP-disease OR	Random dataset split into 50% training set and 50% validation set	No information	No information
**External validation study**														
Tota, 2019 (a, b)	Leave-one-out jackknife	HG, CC	United States	*A priori* selection	2009–2014 (control) and 2011–2015 (case)	1 year	Interview using computer-assisted self-interview, clinical data	OPC	No information	-	No information	No information	116 OPC cases	No information

Abbreviations: GWAS, genome wide association studies; IARC, the international Agency for Research on Cancer; OCC, oral cavity cancer; OPC, oropharyngeal cancer; OR, odds ratio; OSCC, oral squamous cell carcinoma; SNP, single nucleotide polymorphism

[a]Study settings: G, general population; H, hospital-based; HG, a combination between hospital-based cases and population-based controls; HM, a combination between hospital-based cases and mixed (hospital- and population-based) controls

[b]Study types: CC, case-control; Cohort

Table S4.3. Details of genetic risk factors-incorporated model development.

First author, year	Country	Outcome[a]	Selection of SNPs	Development of GRS	Selection of phenotypic factors	Methods of development of combined model
Bao, 2020b	China	OCC	Genetic variations in selenoprotein genes followed by genotype detection and Hardy-Weinberg Equilibrium	Unweighted allele count model	No information	Logistic regression
Chung, 2017	Taiwan	OCC	HapMap database followed by logistic regression	Unweighted allele count model	No information	Logistic regression
Chung, 2019	Taiwan	OCC	Somatic mutations reported in the Cancer Genome Atlas followed by logistic regression	Unweighted allele count model	No information	Cox proportional hazard
Fritsche, 2020a	United Kingdom	OCC	UK Biobank GWAS PheCode	Weighted allele count model; weighted by log odds derived from external GWAS	Firth-corrected logistic regression	Logistic regression
Fritsche, 2020b	United Kingdom	OCC[f]	UK Biobank GWAS FINNGEN	Weighted allele count model; weighted by log odds derived from external GWAS	Firth-corrected logistic regression	Logistic regression
Miao, 2016	China	OCC	Previous reports on miRNA polymorphisms and cancer risk	Unweighted allele count model	No information	Logistic regression

Abbreviations: AIC, Akaike Information Criterion; AUROC, area under the receiver operating characteristic curve; CC, case-control; CI, confidence interval; EHR, electronic health record; EV, external validation; GWAS, genome-wide association study; IARC, the International Agency for Research on Cancer; IV, internal validation; MAF, minor allele frequency; nsCL/P, non-syndromic cleft lip.palate; OCC, oral cavity cancer; O/E, observed/expected; OPC, oropharyngeal cancer; Sens, sensitivity; SNP, single nucleotide polymorphism; Spec, specificity

[a]Each prediction model is for either a single- or combined-outcome.

[b]Classification of prediction model according to the TRIPOD guidelines: 1a, development only; 1b, development and validation using resampling; 2a, random split-sample development and validation; 2b, non-random split-sample development and validation; 3, development and validation using separate data; 4, validation only (not applicable to any of the included models)

[c]Included SNPs: rs1800668, rs3746165, rs7310505, rs4964287, rs9605030, rs3788317, rs13054371

[d]Included SNPs: rs2070833, rs550675, rs139994842, rs2822641

[e]Included SNPs: rs550675, rs28647489

[f]Tongue cancer
