# Supplementary material for: Risk Prediction Models for Oral Cancer: A Systematic Review
Source: Cancers (Basel). 2024 Jan 31;16(3):617. doi: 10.3390/cancers16030617 (PMC10854942; doi:10.3390/cancers16030617)
Supplement: Supplementary file 1 [file cancers-16-00617-s001.zip › Supplementary File Table S5. List of identified SNPs.pdf]

List of identified SNPs in genetic studies

Table S5. List of considered genes and SNPs as the risk factors in the included genetic models.

Author, year	Gene	SNP	Genotypes
Bao, 2020	GPx1	rs1800668	GG
			AG
			AA
			AG + AA
	GPx1	rs3448	T>C
	GPx4	rs3746165	GG
			AG
			AA
			AG + AA
	GPx4	rs4807543	G>T
	TXNRD1	rs7310505	CC
			CA
			AA
			CA + AA
	TXNRD1	rs4964287	CC
			CT
			TT
			CT + TT
	TXNRD2	rs9605030	CC
			CT
			TT
			CT + TT
	TXNRD2	rs3788317	GG
			GT
			TT
			GT + TT
	TXNRD2	rs13054371	TT
			TC
			CC
			TC + CC
	TXNRD1	rs6539137	A>T
	TXNRD1	rs17202060	C>T
Chung, 2019	TP53	rs11652704	C/C
			C/T
			T/T
	TP53	rs12951053	A/A
			C/A
			C/C
	TP53	ls17882227	C/C
			T/C
			T/T
	CASP8	rs6745051	A/A
			C/A
			C/C
	CASP8	rs7608692	A/A
			A/G
			G/G
	CASP8	rs6754084	C/C
			T/C
			T/T
	FAT1	rs28647489	A/A
			G/A
			G/G
	FAT1	rs2306990	C/C
			C/T
			T/T
	FAT1	rs11724817	A/A
			A/T
			T/T
	FAT1	rs2130909	C/C
			T/C
			T/T
	FAT1	rs10009030	A/A
			C/A
			C/C
	FAT1	rs2637777	G/G
			G/T
			TAT
	FAT1	rs10434309	C/C
			C/T
			T/T
	COL9A1	rs550675	C/C
			C/T[a]
			T/T
	NOTCH1	rs201174576	T/T
			G/T[b]
			G/G
Chung, 2017	BRCA1	rs2070833	A/C
	BRCA1	rs3737559	A/G
	BRCA1	rs8176199	A/C
	COL9A1	rs1406844	C/T
	COL9A1	rs3806091	A/G
	COL9A1	rs3828777	A/G
	COL9A1	rs495558	G/T
	COL9A1	rs518558	A/G
	COL9A1	rs544179	A/G
	COL9A1	rs550675	C/T[a]
	COL9A1	rs616621	A/G
	COL9A1	rs679521	C/G
	COL9A1	rs9455009	C/G
	COL9A1	rs9455039	A/T
	COL9A1	rs997953	A/G
	COL9A2	rs1846158	C/T
	COL9A2	rs209918	C/T
	COL9A2	rs2273195	GT
	DNAJA1	rs1097134	A/G
	DNAJA1	rs3758276	C/T
	DNAJA1	rs4879658	C/T
	GDF15	rs1054564	C/G
	HDGFRP	rs1041889	C/T
	HDGFRP	rs1042112	C/T
	HSPA13	rs2822638	C/T
	HSPA13	rs2822641	A/C
	HSPA13	rs2822644	G/T
	HSPA13	rs2822648	A/G
	HSPA13	rs7282521	C/T
	MAOA	rs2072744	A/G
	MAOA	rs2283725	A/G
	MAOA	rs4301558	A/C
	MAOA	rs5906883	A/C
	MMP3	rs522616	A/G
	MMP3	rs605949	C/T
	NOTCH1	rs139994842	A/G
	NOTCH1	rs200699541	C/T
	NOTCH1	rs201174576	G/T[b]
	PTGS2	rs1119064	A/G
	PTGS2	rs689466	A/G
	CHAF1A	rs1165705	G/T
	S100A1	rs3006475	A/C
	SH3GL1	rs243402	G/T
	SH3GL1	rs243404	C/T
	SH3GL1	rs73234	C/G
	UBL5	rs2233678	C/G
	UBL5	rs2233679	C/T
	UBXN6	rs1044510	A/G
	UBXN6	rs932276	A/G
	CHAF1B	rs1130214	G/T
Miao, 2016	miR-146a	rs2910164	GG/GC/CC
	miR-608	rs4919510	AA/AG/GG
	miR-196a2	rs11614913	GG/AG/AA

[a,b]These genotypes were considered more than once.

Note: Two other models that incorporate genetic factors were derived from larger GWAS studies of which their SNPs were not reported. In the study by Fritsche et al., the detail of the risk models is publicly available and accessible through the PRSWeb.
