# Supplementary material for: Risk Prediction Models for Oral Cancer: A Systematic Review
Source: Cancers (Basel). 2024 Jan 31;16(3):617. doi: 10.3390/cancers16030617 (PMC10854942; doi:10.3390/cancers16030617)
Supplement: Supplementary file 1 [file cancers-16-00617-s001.zip › Supplementary File Table S6. Identified models that were not included in the published rapid review.pdf]

Table S6. List of identified articles.

First author, year	Country	Outcome	Title
Antunes, 2013	Brazil	OCC	Joint and independent effects of alcohol drinking and tobacco smoking on oral cancer: a large case-control study
Bao, 2020a	China	OCC	Selenoprotein genetic variants may modify the association between serum selenium and oral cancer risk
Chen, 2017	China	OCC	Dietary score and the risk of oral cancer: a case-control study in Southeast China
Chen, 2022	China	OCC	Association between serum arsenic and oral cancer risk: A case-control study in Southeast China
He, 2021a	China	OCC	Association Between Rare Earth Element Cerium and the Risk of Oral Cancer: A Case-Control Study in Southeast China
He, 2021b	China	OCC	Association Between Rare Earth Element Cerium and the Risk of Oral Cancer: A Case-Control Study in Southeast China
**Genetic models**			
Bao, 2020b	China	OCC	Selenoprotein genetic variants may modify the association between serum selenium and oral cancer risk
Chung, 2017	Taiwan	OCC	Combined Genetic Biomarkers and Betel Quid Chewing for Identifying High-Risk Group for Oral Cancer Occurrence
Chung, 2019	Taiwan	OCC	Variants in FAT1 and COL9A1 genes in male population with or without substance use to assess the risk factors for oral malignancy
Fritsche, 2020a	United Kingdom	OCC	Cancer PRSweb: An Online Repository with Polygenic Risk Scores for Major Cancer Traits and Their Evaluation in Two Independent Biobanks
Fritsche, 2020b	United Kingdom	OCC	Cancer PRSweb: An Online Repository with Polygenic Risk Scores for Major Cancer Traits and Their Evaluation in Two Independent Biobanks
Miao, 2016	China	OCC	Association of microRNA polymorphisms with the risk of head and neck squamous cell carcinoma in a Chinese population: a case-control study
