# Supplementary figures and images for: Risk Prediction Models for Oral Cancer: A Systematic Review
Source: Cancers (Basel). 2024 Jan 31;16(3):617. doi: 10.3390/cancers16030617 (PMC10854942; doi:10.3390/cancers16030617)

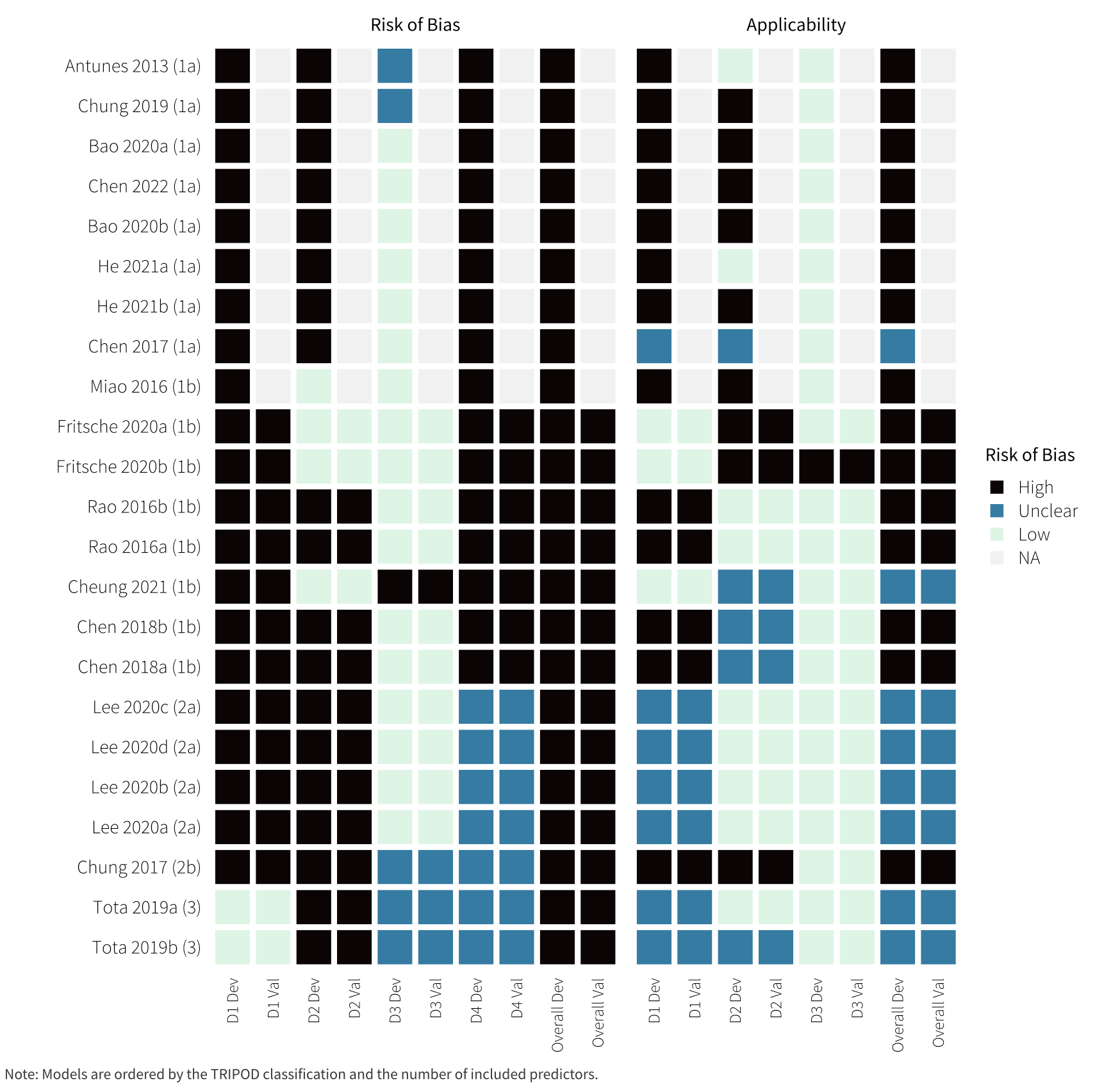

Supplement: Supplementary file 1 [file cancers-16-00617-s001.zip › Supplementary File Figure S1. Risk of bias of the included studies.png]

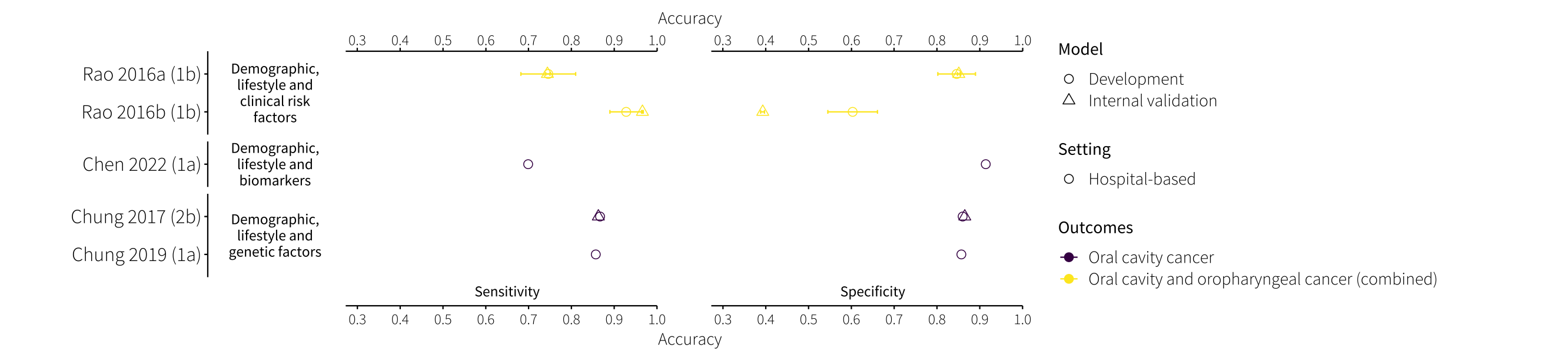

Supplement: Supplementary file 1 [file cancers-16-00617-s001.zip › Supplementary File Figure S2. Reported accuracy of the included models.png]
